# The submerged footprint of Perito Moreno glacier

**DOI:** 10.1038/s41598-020-73410-8

**Published:** 2020-10-02

**Authors:** Emanuele Lodolo, Federica Donda, Jorge Lozano, Luca Baradello, Roberto Romeo, Donaldo M. Bran, Alejandro Tassone

**Affiliations:** 1 Istituto Nazionale di Oceanografia e di Geofisica Sperimentale (OGS), Borgo Grotta Gigante 42/C, 34010 Trieste, Italy; 2 Instituto de Geociencias Básicas, Aplicadas y Ambientales (IGeBA), Ciudad Universitaria, Pabellón II - C1428EHA, CONICET - Universidad de Buenos Aires, Buenos Aires, Argentina

**Keywords:** Geomorphology, Environmental sciences

## Abstract

Perito Moreno is the most famous calving glacier of the South Patagonia Icefield, the largest temperate glacier system of the Southern Hemisphere. Unlike most of the glaciers in the region that have strongly retreated in recent decades, the position of Perito Moreno glacier front remained relatively unchanged in the last century. However, earliest photographic documents show that, at the end of the nineteenth century, the front was *ca.* 800 m behind the current position. There is no reliable information about the positions of the Perito Moreno front in earlier times. Here we show evidence of two subaqueous moraine systems both in the Canal de Los Témpanos and in the Brazo Rico, the two arms of Lago Argentino along which Perito Moreno glacier has flowed over time. These moraines, identified for the first time in the Canal de Los Témpanos from bathymetric and high-resolution seismic profiles, mark the position of the largest glacier advance, tentatively correlated with the moraines of the “Herminita advance” identified and dated onland. We interpret these bedforms as the evidence of the most pronounced advance of Perito Moreno glacier during the mid-Holocene cooling event that characterized this sector of the Southern Hemisphere. This study highlights the importance of subaqueous glacial bedforms, representing decisive records of the glacial history and palaeoclimate, which could help unveiling the origin of the different behavior of glaciers like Perito Moreno that in a warming climate are relatively stable.

## Introduction

In Patagonia there is considerable evidence, provided by studies on moraine systems, landforms and stratigraphic records, that glaciers and icefields have expanded and contracted in the past in response to variations in climate systems^1–3^. In this region, the icefields are nourished by mid-latitude weather systems characterized by abundant precipitation, causing high ablation rates, steep mass-balance gradients and high ice velocities^3^. This is because southern Patagonia lies at the northern edge of the Southern Westerly Winds, an important component of the global atmospheric system that greatly controls the passage of frontal systems, and therefore precipitation, in the mid-latitudes^4^. In addition, Patagonian glaciers react rapidly to even small changes in climate given the temperate setting: this has led Patagonia to contain some of the fastest waning ice masses in the Southern Hemisphere at present^5^. In fact, among global glacier mass changes, the Patagonian icefields reveal the most negative mass changes^6,7^ and are the largest contributors to sea-level rise among the South America glaciers, largely driven by dynamic adjustments of the tidewater and lake calving glaciers^8^. The freshwater outlet glaciers have a distinctive relationship with climate change, mostly due to their unique sensitivity to topography, and the strong correlation between calving rates and water depth at the glacier terminus^9^. There is evidence that calving glaciers have not been responding directly to climate this century, their behavior being more closely controlled by trough geometry and topography than by climate^10^. This is because a strong influence of topography and water depth is expected in influencing ice marginal positions and retreat dynamics^11^. For the case of Perito Moreno, it has been demonstrated that seasonal ice-front variations would be primary due to frontal ablation (subaqueous melting), which is correlated with seasonal lake water temperature variations^12^. The thermal notch that forms at the base of the glacier front is considered the trigger mechanism of calving, abundantly exceeding the magnitude of the ice speed.

**Figure 1:**
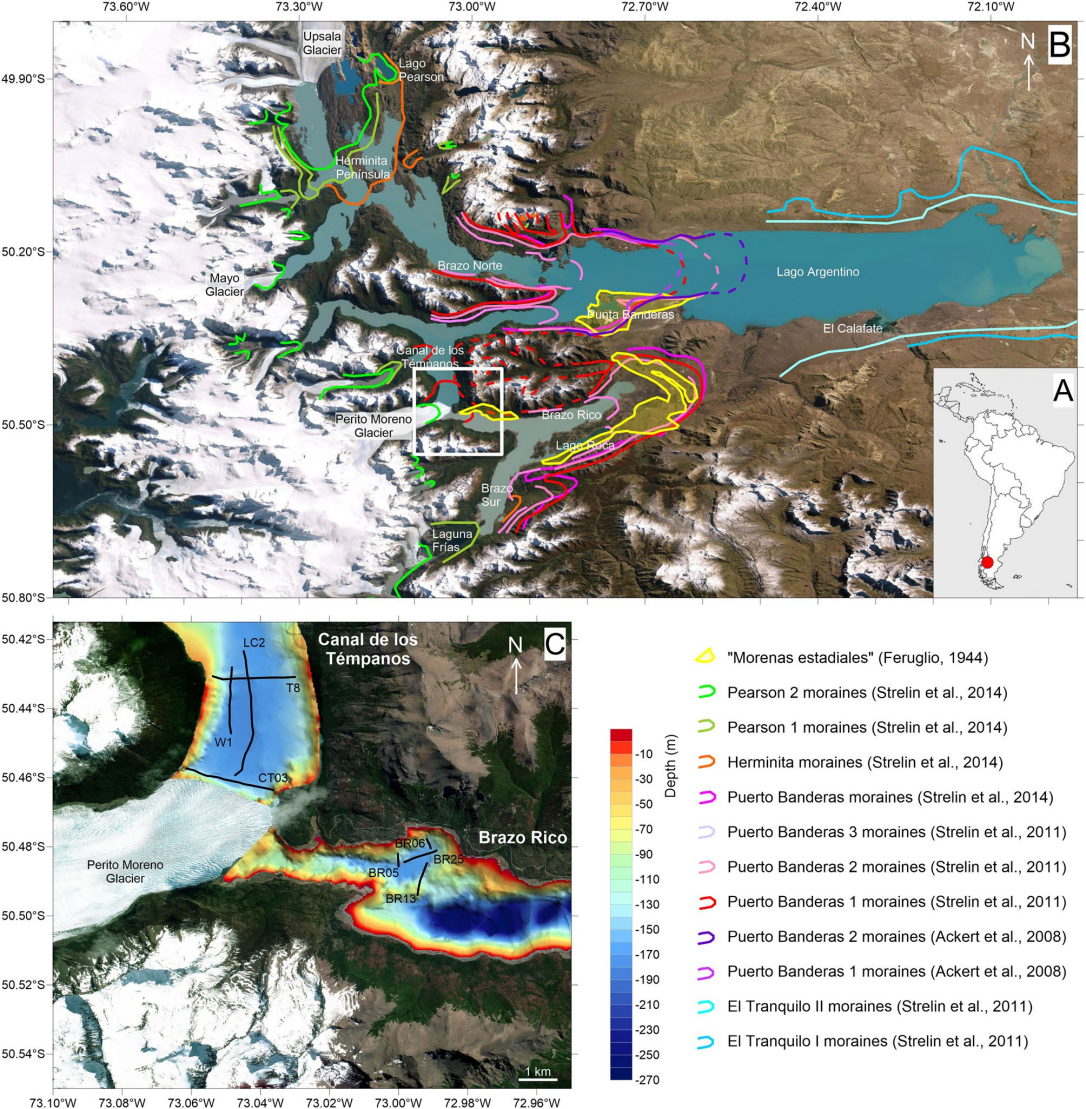
Location map and bathymetry of the study area. (**A**) Map of South America with the study area location indicated by a red dot. (**B**) General map of the Lago Argentino in southern Patagonia with the major outlet glaciers flowing along the deep arms of the lake. The positions of the moraine complexes mapped in the eastern sector of the South Patagonia Icefield have been reported from literature, with the corresponding authors. The base map of this Figure has been taken from GoogleEarth, and was subsequently edited with CorelDraw software. (**C**) Detailed bathymetric map of the southern part of Canal de Los Témpanos and the western part of Brazo Rico, with positions of the seismic profiles presented in Figs. 3 and 4 (thin black lines). The base map of this Figure has been taken from GoogleEarth, and was subsequently edited with CorelDraw software.

Perito Moreno, one of the main eastern outlet glaciers originating from the South Patagonian Icefield, terminates in Lago Argentino at the junction of Brazo Rico and Canal de los Témpanos (Fig. 1). Although most of the Lago Argentino outlet glaciers underwent considerable ice mass loss and retreated during the last 80–100 years, Perito Moreno glacier shows an apparent anomalous behavior and can be regarded as having been stable during at least the last half-century^13,14^. Its annual net accumulation rates of 5465 ± 480 mm w.e./year are among the highest worldwide^15^. The bottom topography of the lake and the high ratio of calving flux to net accumulation explain the remarkable stability of Perito Moreno glacier, which is in contrast to the retreat of other glaciers in this region^16^. Attachment of the calving terminus to topographic pinning points plays a key role in the glacier dynamics, since ice flow is impeded in the central section by the Magallanes Península^10,15^. The stability of Perito Moreno glacier thus points out that anomalous recession or advance can follow once the glacier terminus either recedes behind or advances from such pinning points^9^. This dampens the glacier response to climate change^16^, as the significance of calving can be considerable, both for interpretation of the glacio-climatic record and for prediction of glacier responses to future climate change^9^.

Perito Moreno glacier reached its present position at the end of the nineteenth century after about 800 m of advance, as documented by historical photographs^17^ (see Fig. 6) and, since then, the position of the glacier front has remained relatively unchanged. It reached the Magallanes Península for the first time in 1917^18^. No significant advancing or retreats of the glacier fronts since the early twentieth century have been documented by the analysis of satellite-derived images^19,20^. The glacier advances between June and December, and retreats between December and April, with a seasonal variation in its position of ± 65 m^21^ possibly due to frontal ablation (subaqueous melting), which is correlated with seasonal lake water temperature variations^12^. This leads to recurrent cycles of advance, closure, damming, flood, failure and subsequent retreat of the glacier front. It has registered numerous front advances at irregular intervals since the end of the nineteenth century, whereas over the past decade, it has shown short-term advances and retreats^21^.

The advances of Perito Moreno glacier form a natural ice-dam as its front arrives over the rocky shore of the Magallanes Península (Fig. 1), blocking the water flow from the Brazo Rico to the Canal de Los Témpanos. Spectacular and powerful water cascades form after semi-periodical ice-dam ruptures^18^, the most recent one at the time of publication having occurred on December 28, 2018. Ice-dam formation is related to the annual advance of the front of the glacier, which reveals a seasonal oscillatory cycle, controlled by the interaction of the glacier front with Magallanes Península and Lago Argentino, creating a feedback mechanism of advance, close, open and retreat^21^.

Most of the present landscape of the Patagonia region is the result of the glacial modification during the Pleistocene and Holocene, when the glaciers expanded their fronts towards the east^22–24^. A recent reconstruction of the Patagonia ice-sheet made at 5 kyr intervals, and based on a compilation of available geomorphological data and recalibrated ages^11^ indicate that this region has responded to climate signals observed in Antarctica (e.g., the Antarctic Cold Reversal), and also that variations in the core of the Southern Westerly Winds played a role in driving glacier advance and recession in function of both the timings and their latitudinal locations.

Lago Argentino is a key location to refine the paleoclimate of this region, thanks to the presence of well-preserved glaciogenic landforms and stratigraphic exposures. The last glaciation, known in the area as “El Tranquilo Glaciation”^23,25^, reached its maximum extent at nearly 15.0 cal kyr BP^3^. At around 12,650 cal year BP, Lago Argentino outlet glaciers underwent a deep recessional phase into the Andean Cordillera, followed by repeated episodes of glacier advances and retreats^23,24^. In the early Holocene, glaciers were typically similar in extent as today^24,26^ and warm conditions were predominant, except perhaps from *ca.* 10 to 9.5 kyr, when Southern Annular Mode-like conditions briefly turned persistently negative, as well as between 8 and 7 to ~ 5 kyr, when glacier expansions are documented in southern Patagonia^27^. From ∼ 4 to 2.5 kyr, positive Southern Annular Mode-like conditions typically occurred^27^. A consistent readvance of glaciers for the last two millennia in the Patagonia region is testified by ^10^Be dated moraines on the Herminita Península (ages are 1.2 ± 0.1 kyr; 2.1 ± 0.2 kyr; 1.4 ± 0.1 kyr), and ^10^Be dated moraines from nearby Lago Pearson (ages from 1.6 ± 0.1 and 1.3 ± 0.1 kyr)^5,24^. Finally, readvance of glaciers was inferred by historical documents and field data. On the eastern South Patagonian Icefield, dendrochronology indicates glacier advance to prominent moraines at 1626–1850 AD^28^. On the Herminita Península, a number of moraines dated using ^10^Be yield ca. 0.2–0.6 kyr^5,24^. In the south-western Lago Argentino basin, moraines inside the mid-Holocene limits document an advance of Frías glacier at 0.2–0.5 kyr^5^.

Concerning a more recent glacial history of the study area, the end of the Little Ice Age at *ca.* 1870 AD is marked by tree ring records, indicating a shift toward warmer temperatures at 1875 AD contemporaneous for the ice retreat and dated moraines^29^. Since the Little Ice Age, the glacier area decreased from 28,091 ± 890 to 22,636 ± 905 km^2^ in 2016, which led to the formation of 1,159 new lakes^29^.

The late Pleistocene-early Holocene glacial evolution of the Patagonia glaciers was mostly reconstructed on the basis of terrestrial moraine deposits, while little information is available on the lake-floor morphology. This is particularly true for the glaciers that now flow into Lago Argentino. Recently, Lodolo et al*.*^30^ and Lozano et al*.*^31^, using bathymetric records and high-resolution seismic profiles, have unveiled the submerged glacial-related deposits along the two southern arms of Lago Argentino, i.e. Brazo Rico ad Brazo Sur, some of which being correlated to those known onland. To date, no moraine bodies have been identified within the Canal de Los Témpanos. The spatial distribution, shape and internal geometries of the moraines are essential elements for reconstructing the dynamic history of glaciers, and in many cases they allow to discriminate the advancement phases from the recessional ones on the basis of the morphologies and the internal structure of the moraines that can be deduced from seismic data.

Here, for the first time, we document the presence of a frontal moraine on the lake-floor that testifies a significant advance of Perito Moreno glacier, which predates that documented by available photos taken at the end of the nineteenth century. The position of the Perito Moreno glacier front before this date, and its possible fluctuations, cannot be confidently reconstructed, because glacier fluctuations overrode almost all older moraines. The morphological characteristics of the flat-bottom trough within the Canal de Los Témpanos along which the glacier has flowed (see Fig. 3), testify that there have been no more recent fluctuations since this major advance. The position of the frontal moraine on the lake-floor coincides with that of the only moraine identified and mapped on land, i.e. the so-called Herminita moraine, even if we do not have direct constraints for attributing its age.

## Results

**Figure 2:**
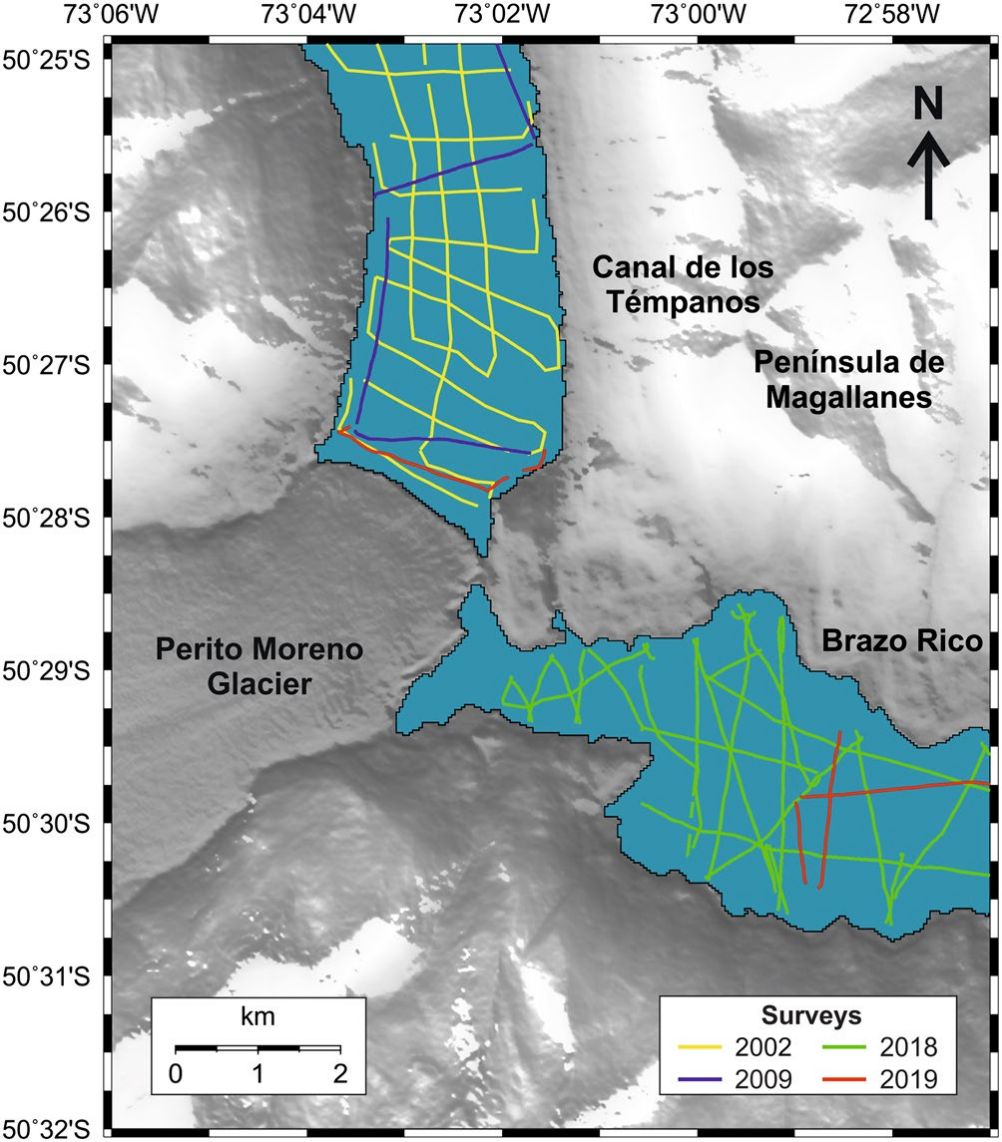
Seismic lines location map. Segments indicate the seismic and bathymetric profiles used in this study, differentiated by colors as indicated in the legend.

**Figure 3:**
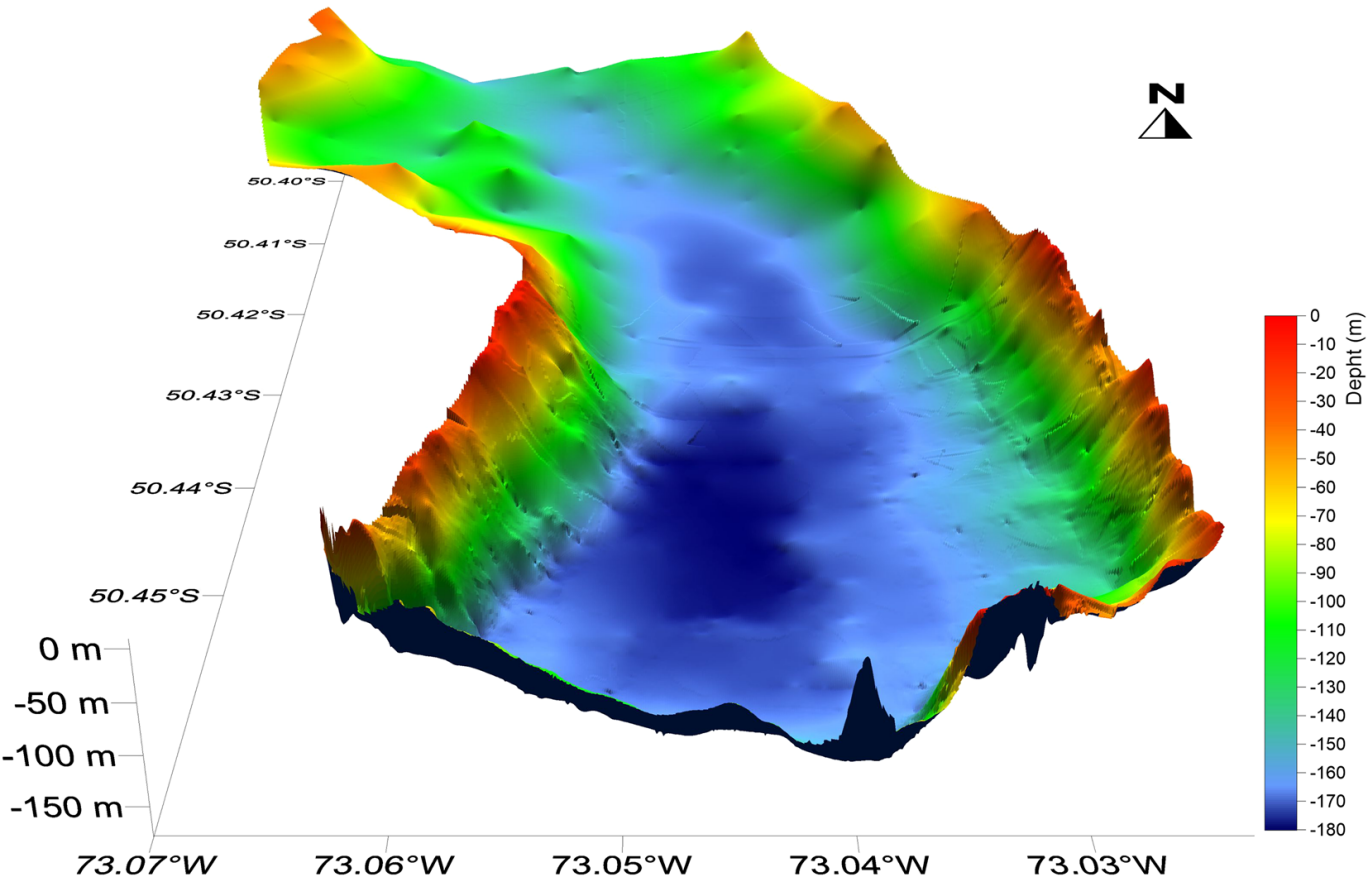
Perspective view of the southern part of Canal de Los Témpanos. 3D shaded-relief image of the lake-floor obtained from available bathymetric data, showing the horseshoe-shaped depression interpreted as a glacial trough.

**Figure 4:**
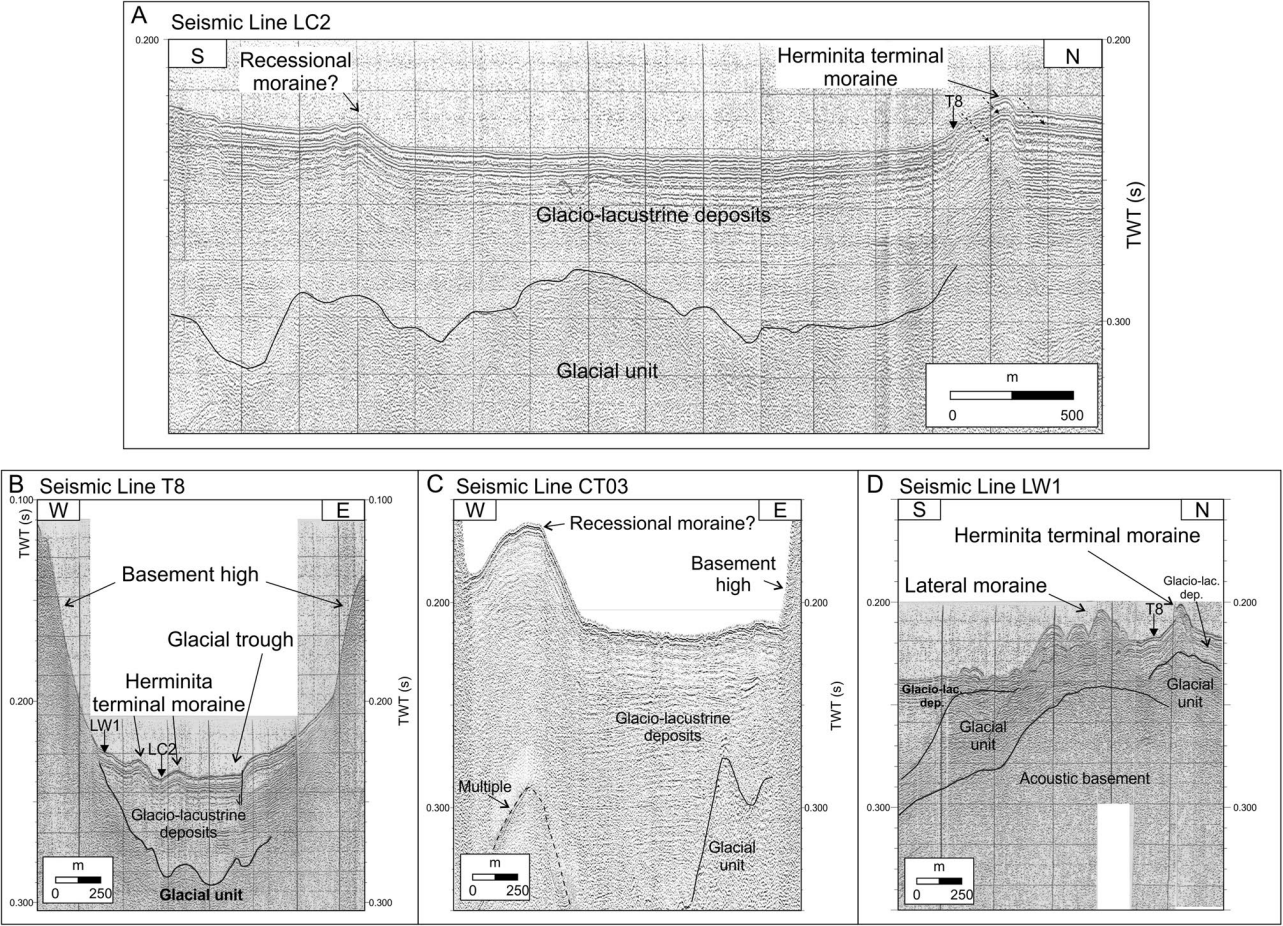
Seimic profiles. High-resolution seismic profiles acquired in the southern part of Canal de Los Témpanos (see Fig. 1 for locations) showing the expression of the subaqueous moraines identified at the lake-floor. Dashed arrows show the occurrence of “ringing” spurios events in the seimic data. Labelled arrows indicate the crossing points of the seismic lines. Stratigraphic units indicated in the profiles are those described and individuated after Lodolo et al*.*^30^.

**Figure 5:**
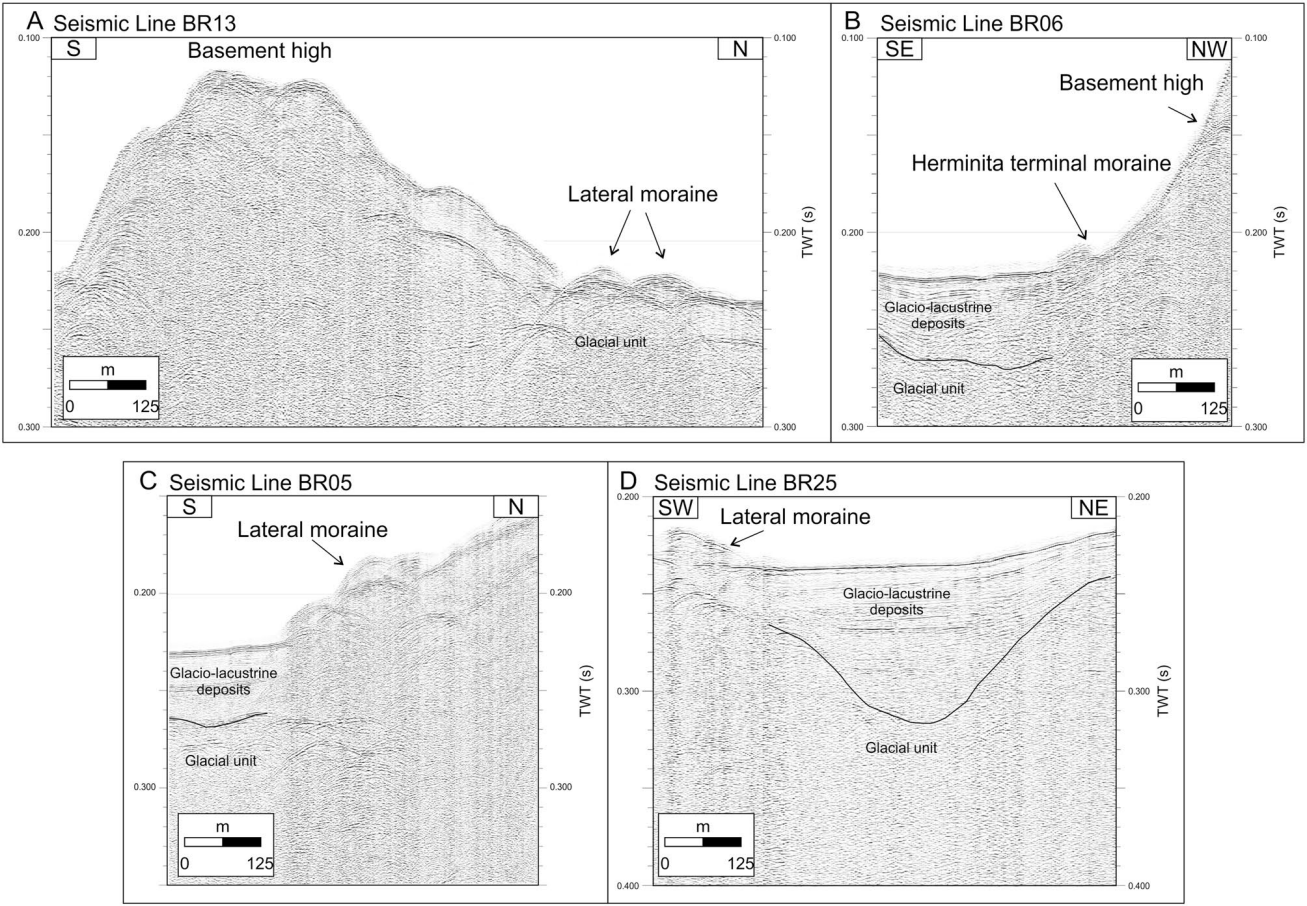
Seimic profiles. High-resolution seismic profiles acquired in the western part of Brazo Rico (see Fig. 1 for locations) showing the expression of the subaqueous moraines identified at the lake-floor. Stratigraphic units indicated in the profiles are those described and individuated after Lodolo et al.^30^.

Bathymetric and high-resolution seismic profiles presented in this paper were acquired during a series of surveys performed in the last two decades (Fig. 2). Studies on the seismostratigraphic and depositional architecture of the study area^30,31^ and on the distribution of the subaqueous landforms highlighting the late-glacial glacier fluctuations^32^ have been already published. They revealed the occurrence of three main seismostratigraphic units^30,31^. The lowermost seismic reflector detectable from seismic profiles is represented by a highly irregular surface, which we interpreted as the top of the acoustic basement, and characterized by a medium- to high-amplitude, chaotic, reflection-free seismic response (Fig. 4D). Above of it, a seismic facies showing a chaotic acoustic response, with an overall irregular morphology and covering the lower, rough basement morphology, is interpreted as an old glacial-related deposit (“glacial unit” in Figs. 4, 5). The uppermost seismic facies is represented by a more than 100-m-thick package of reflectors (Fig. 4C) that follow the lower, rough morphology (Fig. 5B), often onlapping it (Figs. 4, 5C, 5D). The overall internal architecture of this seismic facies is well-layered and made by sub-horizontal, sub-parallel, and continuous reflectors; it has been interpreted as a glacio-lacustrine deposit^30,31^. Here we focus our analysis on the moraine complex closest to the glacial front found from the geophysical data. Among the several subaqueous moraine ridges identified on the lake-floor along both Brazo Rico and Canal de Los Témpanos, the closest glaciogenic bedforms to the present-day Perito Moreno front are represented by two moraine systems which border two almost flat lake-floor depressions, *ca.* 170 m deep (Fig. 1C). In the Canal de Los Témpanos such a depression is more easily identifiable due to the flat-lying morpho-bathymetric character of this part of the lake-floor (Fig. 3). This horseshoe-shaped depression trending along the Canal de los Témpanos is *ca.* 3500 m long and 400–1200 m wide, in the most distal and most proximal position, respectively, with respect to the glacier terminus. We suggest that the depression represents a glacial trough. Our hypothesis is supported by the occurrence of an asymmetric sedimentary ridge at its distal boundary (Fig. 4), which we interpret as a frontal moraine. It lies at 3500 m from the present-day glacier front, is *ca.* 12 m high (note that the time to depth conversion has been obtained applying a water sound velocity of 1432 m/s, according to Lodolo et al.^30^) and reveals a longer, gentler slope towards south, with respect to the steepest, shorter flank to the north. Internally it is constituted by discontinuous reflectors, apparently inclined toward south, according with the surface morphology. Toward south, at a distance of *ca.* 2500 m from this frontal moraine, another sediment ridge is recognizable on the lake-floor. It is *ca.* 6 m high, is almost symmetrical and reveals an apparent internal stratification. We interpret this feature as a recessional moraine, although the lack of additional seismic data prevents us to constrain our hypothesis. The western flank of the trough is bounded by highly irregular relieves, up to *ca.* 30 m high, almost parallel to the western lake shore. Internally, they are constituted by irregular, medium- to high-amplitude reflectors, revealing mound-shaped features, with frequent diffraction hyperbolae (Fig. 4). We interpret these sediment ridges as lateral moraines, possibly formed during several glacier overriding stages, including the last, major one, marked by the glacial trough. In the more proximal area to the outlet glacier, at *ca.* 200 m from the glacier front, a 28 m high mounded body characterized by an apparent stratified seismic facies has been deposited (Fig. 4C). Its internal reflector configurations resemble those characterizing the recessional moraines described by Lodolo et al.^30^, and thus we interpret such a feature accordingly.

**Figure 6:**
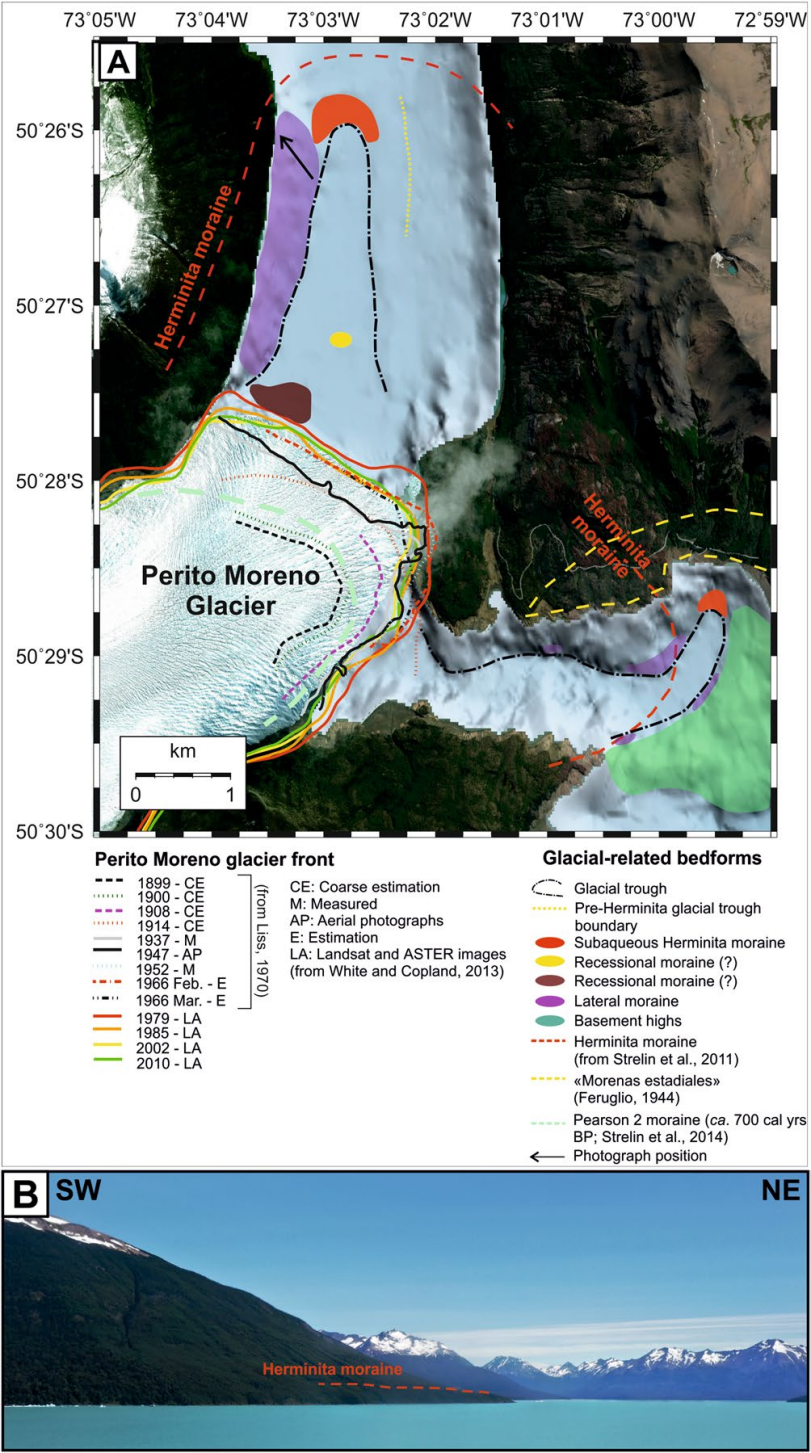
Geomorphology of the study area and former positions of the Perito Moreno glacier front. (**A**) Geomorphological map of the study area, showing the location of the subaqueous moraine bodies identified from the geophysical data analysis. The external limits of the frontal positions of the Perito Moreno glacier derived from historical photographs, satellite-derived optical images and aerial photos, have also been included, so as the position of the Herminita moraines (see legend for further details). The base map in this Figure has been taken from GoogleEarth, and was subsequently edited with CorelDraw software. (**B**) The western shoreline of the Canal de Los Témpanos where the slope break indicated by the red hatch represents the top part of the Herminita lateral moraine. Photograph taken by one of the authors (J. Lozano).

The westernmost sector of Brazo Rico presents a more complex bathymetry than that of the southern sector of the Canal de Los Témpanos, and there are various morphological highs that reflect in many places the surrounding topography (Fig. 5). The closest frontal moraines to the present-day terminus of Perito Moreno glacier lie at the termination of a northward bend of a *ca.* 900 m wide, horseshoe-shaped ridge. Here, at a distance of 3700 m from the glacier front, the lake bathymetry reveals a depression similar to that identified in the Canal de Los Témpanos, which we interpret as a glacial trough (Fig. 5). It bends toward NE creating a sort of embayment of the lake shore. To the east, the trough appears to be bounded by a ridge with *ca.* 50 m of relief composed by glaciogenic deposits (Fig. 5). Moraines in Brazo Rico are constituted by a series of sediment bodies, ranging from a few to *ca.* 15 m in height, internally characterized by a series of diffractions and spurious reflections. They occur both as isolated features deposited on the lake-floor, and within morphological relieves, overlying older glacial deposits, and revealing an overall irregular convex shape (Fig. 5). Since such sediment bodies bound almost completely the glacial trough, we suggest they represent lateral moraines. Figure 6 illustrates the lake-floor morphological map of this sector of the Lago Argentino derived from the data analysis.

## Discussion

In the eastern sector of the South Patagonia Icefield more proximal to Perito Moreno glacier, there are two key areas in which the moraines have been extensively mapped and dated, i.e. in the surroundings of the Upsala glacier (Herminita Península and Lago Pearson), and in the outwash plain of the Frías glacier (Laguna Frías)^5,27^. These locations constitute the most thorough records of directly dated glacier fluctuations since late-glacial time in the region of Lago Argentino^33^. Exposure ages range from 5.2 ± 0.4 to 4.6 ± 0.4 kyr on the outer Pearson moraines near Lago Argentino, and from 5.7 ± 0.5 to 4.5 ± 0.4 kyr on the Herminita Península^5^. More to the south, at Laguna Frías, ages range from 6.1 to 5.7 kyr^5,24^. There are no ^10^Be dated Holocene moraines prior to about 6 kyr, with a possible exception of two older ^10^Be ages (8660 ± 180 and 6990 ± 200 year BP) on small ridges preserved along the uppermost left lateral at Lago Pearson^27^. In general, these data show glacier expansions at 6100 ± 390, 4450 ± 220, 2300–2000, 1400 ± 110, 600–500, 360 ± 30, and 240 ± 20 year BP^33^. Along the steep flanks bordering the Canal de Los Témpanos, the only presence of terrestrial moraines was reported by Strelin et al*.*^23^, and was associated to the so-called “Herminita advance” (see Fig. 16 of Strelin et al*.*^23^ and Fig. 1 of this work). As stated above, the Herminita moraines pertaining to this advance were identified in the southern sector of the Herminita Península, and described for the first time by Malagnino and Strelin^34^. Strelin et al*.*^23^ assigned a late-glacial age to the Heminita moraines, whereas Aniya and Sato^35^ attributed these glacial-related bedforms to the second-oldest Neoglacial advance (*ca.* 2340–2120 cal year BP). The most recent studies however, have shown that the Herminita moraines are mid-Holocene in age^5,27,33^, as the ^10^Be ages derived from exposed boulders are significantly younger with respect to the radiocarbon measurements. To reconstruct the glacier behavior during the earliest Holocene prior to ~ 6 ka, most of the authors have used the complementary ^14^C chronology presented in Strelin et al*.*^23,24^, which include maximum-limiting ages on reworked pieces of wood in sediments in stratigraphic sections and on samples from basal sections of bog cores around Laguna Frías^23,24^. These data indicate that after the late-glacial advances, the South Patagonia Icefield retreated close to present ice margins^23,24^. Any early Holocene advances (e.g., ~ 8–7 kyr in Strelin et al., 2014) of the glacier margin were within the subsequent maxima expansions. The pre-6000 yr BP events not preserved at Lago Argentino would reflect climatic events within a generalized warm period on a scale too brief for the much larger South Patagonia Icefield to respond and leave a preserved moraine record^33^. No age information are presently available on the moraine deposits along the steep walls of Canal de Los Témpanos.

The locations of the subaqueous frontal moraines identified in this work in the two eastern arms of Lago Argentino have been correlated with those identified and mapped onshore by Strelin et al.^23^. Our interpretation is not based on specific dating performed on the moraine deposits found at the lake-floor, but essentially it is based on their geographical location, which coincides with that of the only moraines identified and mapped on land, with the limits of the age uncertainties described above. According to our interpretation, the lake-floor bedforms described in this study may represent the submerged expression of the “Herminita advance” identified by Strelin et al*.*^23^ and recently dated as Mid Holocene^5,27,33^, thus recording the latest major re-advance of Perito Moreno glacier in the last 6000 years.

Considering that no information is available on the position of the glacier front before the end of the nineteenth century, our study contributes to unveil the Perito Moreno dynamics since the mid-Holocene. The almost featureless lake-floor within the glacial-related depression bounded by frontal and lateral moraines testifies that, after the “Herminita advance”, Perito Moreno glacier has not undergone other similar advances. During its retreat, the Perito Moreno probably stood still for the period of time necessary to build the recessional moraine located at a distance of about 1000 m from the current glacier front (Figs. 4A, 6). In addition, the glacial trough appears to occupy the central-western side of Canal de Los Témpanos. This evidence, together with the occurrence of well-developed lateral moraines along the western side of the glacial depression would suggest that, at least during the “Herminita advance”, the glacier flowed and/or was partly grounded only in this part of the Canal de Los Témpanos, instead of occupying it completely. The eastern edge of the glacial depression may have been set along a N–S oriented fault, as available seismic lines would suggest (Fig. 4). The pronounced recessional moraine located at a distance of *ca.* 200 m from the present-day grounding line would then possibly record a prolonged stillstand phase of the Perito Moreno glacier, which could be related to the deep glacier recession after the Herminita moraine advance^23,24^. Pinning points may have played an important role in stabilizing the glacier termini at least in late-Holocene time^24^.

It is possible that moraines of the mid-Holocene are much more widespread in Patagonia, but the limited number of studies performed and the possibility that these have been re-occupied by glaciers make these reconstructions very uncertain^11^. Similar mid-Holocene advances have been observed at this time in the sub-Antarctic islands^36^ and in New Zealand^37,38^. Some authors^5^ suggested that the 6–4 kyr mid-Holocene advance was due to a northward expansion of the Southern Westerly Winds at this time. These mid-Holocene glacier expansions were followed by warming and glacier recession to similar positions to the present^39^.

It is important to underline, however, that while the reconstructions of glacial dynamics up to the late-glacial are fairly well delineated, it is extremely difficult to distinguish the more recent moraines from those of the Holocene and late Pleistocene, as claimed by various authors. Undoubtedly, a systematic multidisciplinary work should be done to reconstruct in greater detail the glacial dynamics of this time span. In this general scenario, our study represents a further step to decipher the complex glacial dynamics of southern Patagonia, and highlights the key role of subaqueous glacial bedforms as signatures of the glacier imprints within lakes. Glacial and glacio-lacustrine deposits thus represent decisive records of the glacial history and paleoclimate and could help unveiling the origin of the different behavior of glaciers like the Perito Moreno which, in a warming climate, is relatively stable.

## Methods

### High-resolution seismic profiles

High-resolution seismic and bathymetric profiles presented here were acquired in the two eastern arms of Lago Argentino between November 2017 and April 2019, for a total length of 442 km of data. These data were complemented with some high-resolution profiles collected by us in 2009 in the Canal de los Témpanos. To integrate this information for the southern sector of the Canal de los Témpanos, we have used some high-resolution seismic and bathymetric profiles acquired in 2002 the frame of a scientific project coordinated by P. Skvarca and M. Paterlini. All seismic data were acquired with a Boomer source composed of an electrodynamic plate powered by a group of capacitors connected to a 3.5 kW generator, and mounted on a catamaran. The generated pulse (400–6000 Hz with a dominant frequency of 2000 Hz) provides submetric resolution. The receiving system was a multichannel streamer composed of eight pre-amplified hydrophones distributed over an active section of 22 m. The streamer used for the surveys performed in 2002 was a single channel streamer composed by eight hydrophones, applying a band-pass filter of 800–3000 Hz. Data acquired during this survey were recorded only on chemical paper (EG&G thermal plotter), and subsequently corrected for both the time scale and horizontal length in order to produce a final version to be presented in this article (see profiles in Fig. 4). The positioning of the collected tracks (in the WGS84 geodetic reference frame) was obtained with a D-GPS interfaced with the acquisition system. Sampling rate was 0.125 ms, shot frequency was 3 shots every 2 s, and record length was 600 ms, acquiring the data with an average boat speed of *ca.* 4 knots. In general, the maximum acoustic penetration was about 120 m below the lake-floor with *ca.* 0.2 m of vertical resolution. Seismic data processing to produce the final seismic images (for profiles acquired during the 2009, 2018 and 2019 surveys) included the following steps: (1) electrical disturbances and noise attenuation by averaging the amplitudes and frequencies on temporal windows of groups of adjacent traces; (2) spherical divergence correction and automatic gain control (with a 10–20 ms windows); (3) band-pass filters applied according to the spectral characteristics of the individual profiles; (4) velocity analysis using a constant velocity stack algorithm; (5) normal move out and stack; and (6) Kirchhoff time migration.

### Bathymetric profiles

The bathymetric map presented in Fig. 1, which was the basis for the identification of the moraine bodies along with the seismic profiles, was created by combining both echo-sounder bathymetric profiles and seismic-derived bathymetric profiles. The bathymetry of the southern part of the Canal de los Témpanos was primarily obtained using profiles acquired in 1999 and available on paper. These data have been geo-referenced and subsequently digitized in the WGS84 reference frame and complemented with some high-resolution seismic profiles acquired in 2009. The bathymetry of the Brazo Rico and Brazo Sur has been obtained by digitizing the first arrival on all the available seismic data in these two sectors, and the resulting two-way travel time (TWT) has been converted in water depths, applying the water lake sound velocity of 1432 m/s^40^. Considering that the seismic surveys were performed in different periods, and that the Brazo Rico/Brazo Sur lake levels were different (higher with respect to the Canal de los Témpanos) due to the ice-damming of Perito Moreno glacier, we leveled all the profiles at the same datum of the Canal de los Témpanos and Lago Argentino. This was achieved by analyzing the difference in time at the intersections among all the seismic lines, and the obtained value was then subtracted to bring it back to the lake level of the Canal de los Témpanos. The final bathymetric map was obtained by applying the Kriging interpolation technique (with a radius of 50 m), which considers both the distance and the degree of variation between known data points when estimating values in unknown areas. This method proved to be the best compared with the other interpolation methods, considering the sparse distribution of our profiles and the distance between the various lines acquired.

## References

[1] Mercer JH (1968). Variations of some Patagonian glaciers since the Late-Glacial. Am. J. Sci..

[2] Hulton NRJ, Purves RS, McCulloch RD, Sugden DE, Bentley MJ (2002). The last glacial maximum and deglaciation in southern South America. Quat. Sci. Rev..

[3] Glasser NF, Harrison S, Winchester V, Aniya M (2004). Late Pleistocene and Holocene palaeoclimate and glacier fluctuations in Patagonia. Glob. Planet. Change.

[4] Toggweiler JR, Russell JL, Carson SR (2006). Midlatitude westerlies, atmospheric CO_2_, and climate change during the ice ages. Paleoceanography.

[5] Kaplan MR (2016). Patagonian and southern South Atlantic view of Holocene climate. Quat. Sci. Rev..

[6] Zemp M (2020). Global glacier mass changes and their contributions to sea-level rise from 1961 to 2016. Nature.

[7] Dussailant I (2020). Two decades of glacier mass loss along the Andes. Nat. Geosci..

[8] Braun MH (2019). Constraining glacier elevation and mass changes in South America. Nat. Clim. Change.

[9] Warren C, Aniya M (1999). The calving glaciers of southern South America. Glob. Planet. Change.

[10] Warren C, Sudgen D (1993). The Patagonian Icefields: a glaciological review. Arctic Alpine Res..

[11] Davies BJ (2020). The evolution of the Patagonian Ice Sheet from 35 ka to the present day (PATICE). Earth Sci. Rev..

[12] Minowa M, Sugiyama S, Sakakibara D, Skvarca P (2017). Seasonal variations in ice-front position controlled by frontal ablation at Glaciar Perito Moreno, the Southern Patagonia Icefield. Front. Earth Sci..

[13] Skvarca P, Naruse R (1997). Dynamic behavior of Glaciar Perito Moreno, southern Patagonia. Ann. Glac..

[14] Skvarca P, Naruse R (2006). Overview of the ice-dam formation and collapse of Glaciar Perito Moreno, southern Patagonia, in 2003/04. J. Glaciol..

[15] Stuefer M, Rott H, Skvarca P (2017). Glaciar Perito Moreno, Patagonia: climate sensitivities and glacier characteristics preceding the 2003/04 and 2005/06 damming events. J. Glaciol..

[16] Rott H, Stuefer M, Siegel A, Skvarca P, Eckstaller A (1998). Mass fluxes and dynamics of Moreno Glacier, Southern Patagonia Icefield. Geophys. Res. Lett..

[17] Liss CD (1970). Morenogletcher in der Patagonischen Kordillere. Zeitschrift für Gletschekunde und Glazialgeologie.

[18] Guerrido CM, Villalba R, Rojas F (2014). Documentary and tree-ring evidence for a long-term interval without ice impoundments from Glaciar Perito Moreno, Patagonia, Argentina. Holocene.

[19] White A, Copland L (2013). Spatial and temporal variations of glacier extent across the Southern Patagonian Icefield since the 1970s. Cryosphere Discuss..

[20] Foresta L (2018). Heterogeneous and rapid ice loss over the Patagonian Ice Fields revealed by CryoSat-2 swath radar altimetry. Remote Sens. Environ..

[21] Lenzano MG (2018). Analyzing the oscillations of the Perito Moreno Glacier, using time-lapse image sequences. Cold Reg. Sci. Technol..

[22] Rabassa J, Coronato A, Martinez O (2011). Late Cenozoic glaciations in Patagonia and Tierra del Fuego: an updated review. Biol. J. Linn. Soc..

[23] Strelin JA, Denton GH, Vandergoes MJ, Ninnemann US, Putnam AE (2011). Radiocarbon chronology of the late-glacial Puerto Bandera moraines, Southern Patagonian Icefield, Argentina. Quat. Sci. Rev..

[24] Strelin JA, Kaplan MR, Vandergoes MJ, Denton GH, Shaefer JM (2014). Holocene glacier history of the Lago Argentino basin Southern Patagonian Icefield. Quat. Sci. Rev..

[25] Strelin J. A. & Malagnino, E. C. Glaciaciones Pleistocenicas del Lago Argentino y Alto Valle del Rio Santa Cruz. *XIII Congreso Geológico Argentino y III Congreso de Exploración de Hidrocarburos Actas* **IV,** 311–325 (1996).

[26] Menounos B (2013). Latest Pleistocene and Holocene glacier fluctuations in southernmost Tierra del Fuego, Argentina. Quat. Sci. Rev..

[27] Kaplan MR (2020). Holocene glacier behavior around the northern Antarctic Peninsula and possible causes. Earth Planet. Sci. Lett..

[28] Masiokas MH (2009). Glacier fluctuations in extratropical South America during the past 1000 years. Palaeogeogr. Palaeocol..

[29] Meier WJ-H, Griessinger J, Hochreuther P, Braun MH (2018). An updated multi-temporal glacier inventory for the Patagonian Andes with changes between the Little Ice Age and 2016. Front. Earth Sci..

[30] Lodolo E (2020). Late-glacial fluctuations of two southern Patagonia outlet glaciers revealed by high-resolution seismic surveys. Quat. Res..

[31] Lozano J (2020). Depositional setting of the southern arms of Lago Argentino (southern Patagonia). J. Maps.

[32] García J, Hall B, Kaplan M, Vega R, Strelin J (2014). Glacial geomorphology of the Torres del Paine region (southern Patagonia): implications for glaciation, deglaciation and paleolake history. Geomorphology.

[33] Reynhout SA (2019). Holocene glacier fluctuations in Patagonia are modulated by summer insolation intensity and paced by Southern Annular Mode-like variability. Quat. Sci. Rev..

[34] Malagnino, E. C. & Strelin, J. A. Variations of Upsala Glacier in southern Patagonia since the late Holocene to the present. in *Glaciological Researches in Patagonia* (eds. Naruse, R. & Aniya, M.) 61–85 (Japanese Society of Snow and Ice, 1992).

[35] Aniya M, Sato H (1995). Morphology of Ameghino Glacier and landforms of Ameghino Valley, southern Patagonia. Bull. Glacier Res..

[36] Hall BL (2009). Holocene glacial history of Antarctica and the sub-Antarctic Islands. Quat. Sci. Rev..

[37] Bravo C (2015). Modelled glacier equilibrium line altitudes during the mid-Holocene in the southern mid-latitudes. Clim. Past Discuss..

[38] Shaefer JM (2009). High-frequency Holocene glacier fluctuations in New Zealand differ from the northern signature. Science.

[39] Moreno PI (2018). Onset and evolution of southern annular mode-like changes at centennial timescale. Sci. Rep..

[40] Zanolla C (2011). Bathymetric map of Lago Fagnano (Tierra del Fuego Island). Boll. Geofis. Teor. Appl..

